# GHS-R1a Deficiency Alleviates Depression-Related Behaviors After Chronic Social Defeat Stress

**DOI:** 10.3389/fnins.2019.00364

**Published:** 2019-04-17

**Authors:** Li Guo, Minglu Niu, Jie Yang, Li Li, Shuhan Liu, Yuxiang Sun, Zhishang Zhou, Yu Zhou

**Affiliations:** ^1^Department of Physiology and Pathophysiology, School of Basic Medical Sciences, Qingdao University, Qingdao, China; ^2^Department of Physiology, Binzhou Medical University, Yantai, China; ^3^Department of Clinic Laboratory, PKU Care Luzhong Hospital, Zibo, China; ^4^Dongying No.1 Middle School, Dongying, China; ^5^Department of Nutrition and Food Science, Texas A&M University, College Station, TX, United States; ^6^Department of Pharmacology, School of Pharmacy, Qingdao University, Qingdao, China; ^7^Institute of Brain Sciences and Related Disorders, Qingdao University, Qingdao, China

**Keywords:** chronic social defeat stress, GHS-R1a, ghrelin, anxiety, depression, mice, behaviors

## Abstract

Ghrelin is an important orexigenic hormone that regulates feeding, metabolism and glucose homeostasis in human and rodents. Ghrelin functions by binding to its receptor, the growth hormone secretagogue receptor 1a (GHS-R1a), which is widely expressed inside and outside of the brain. Recent studies suggested that acyl-ghrelin, the active form of ghrelin, is a persistent biomarker for chronic stress exposure. However, how ghrelin/GHS-R1a signaling contributes to stress responses and mood regulation remains uncertain. In this study, we applied the chronic social defeat stress (CSDS) paradigm to both *GHS-R1a* knock-out (*Ghsr*^-/-^) mice and littermate control (*Ghsr*^+/+^) mice, and then measured their depression- and anxiety-related behaviors. We found that *Ghsr*^+^*^/^*^+^ mice, but not *Ghsr*^-/-^ mice, displayed apparent anxiety and depression after CSDS, while two groups mice showed identical behaviors at baseline, non-stress state. By screening the central and peripheral responses of *Ghsr*^-/-^ mice and *Ghsr*^+/+^ mice to chronic stress, we found similar elevations of total ghrelin and adrenocorticotropic hormone (ACTH) in the serum of *Ghsr*^-/-^ mice and *Ghsr*^+/+^ mice after CSDS, but decreased interleukin-6 (IL-6) in the serum of defeated *Ghsr*^-/-^ mice compared to defeated *Ghsr*^+/+^ mice. We also found increased concentration of brain derived neurotropic factor (BDNF) in the hippocampus of *Ghsr*^-/-^ mice compared to *Ghsr*^+/+^ mice after CSDS. The basal levels of ghrelin, ACTH, IL-6, and BDNF were not different between *Ghsr*^-/-^ mice and *Ghsr*^+/+^ mice. Our findings thus suggested that the differential expressions of BDNF and IL-6 after CSDS may contribute to less anxiety and less despair observed in GHS-R1a-deficient mice than in WT control mice. Therefore, ghrelin/GHS-R1a signaling may play a pro-anxiety and pro-depression effect in response to chronic stress, while GHS-R1a deficiency may provide resistance to depressive symptoms of CSDS.

## Introduction

Ghrelin is a 28-amino-acid peptide hormone which is principally synthesized in the stomach and normally associated with feeding behavior and energy homeostasis (Kojima et al., 1999). Although there is no clear evidence of ghrelin neurons in the brain (Furness et al., 2011), circulating ghrelin in acylated form is capable to cross the blood-brain barrier and binds to central ghrelin receptors (growth hormone secretagogue receptor 1a, GHS-R1a) via active transport and direct diffusion (Schaeffer et al., 2013; Cabral et al., 2014). GHS-R1a is widely distributed throughout the brain, not only in traditional feeding and metabolism-associated regions like arcuate nucleus (ARC) (Zigman et al., 2006; Mani et al., 2014), but also in stress response and emotion-associated areas such as the amygdala, prefrontal cortex (PFC) and the hippocampus (Alvarez-Crespo et al., 2012; Spencer et al., 2012; Hornsby et al., 2016; Harmatz et al., 2017), and in reward centers like ventral tegmental area (VTA) as well (Abizaid et al., 2006; Muller et al., 2015). Recent studies highlight that ghrelin and GHS-R1a play complex roles in the regulation of a diverse number of brain functions, including hunger and metabolism, learning and memory, reward and addiction, motivation, stress responses, anxiety, and depression (Muller et al., 2015; Spencer et al., 2015).

A recent study suggested that acyl-ghrelin is not only a “hunger” hormone but also a persistent biomarker for chronic stress exposure (Yousufzai et al., 2018). Indeed, acyl-ghrelin level have been shown to remain elevated in C57BL/6 mice after chronic social defeat stress (CSDS) (Lutter et al., 2008), in rats after chronic immobilization stress (Meyer et al., 2014; Harmatz et al., 2017), and in mice after chronic unpredictable mild stress as well (Huang et al., 2017). Meanwhile, the prolonged increase in circulating level of acyl-ghrelin was observed not only in adult or adolescent rodents, but also in vulnerable adolescent humans exposed to chronic severe stressor (Meyer et al., 2014; Harmatz et al., 2017; Yousufzai et al., 2018). Although elevation of circulating acyl-ghrelin induced by chronic, psychology stress is always accompanied by exacerbated anxiety- and depression-like behaviors, the reason why chronic stress induces ghrelin release and how ghrelin contributes to stress responses and mood regulation remains uncertain. Previous studies suggested that ghrelin may play a dual role in anxiety and depression. For example, ghrelin was reported to both promote (Carlini et al., 2002, 2004; Hansson et al., 2011; Currie et al., 2012; Spencer et al., 2012; Meyer et al., 2014) and alleviate (Lutter et al., 2008; Spencer et al., 2012; Jensen et al., 2016; Huang et al., 2017) anxiety- and depression-like behaviors, a dual but disparate effect which may depends on both contextual states (for example, non-stress vs. stress, acute vs. chronic stress, mild vs. strong stress) and physiological states (for example, food availability) of the animal (Spencer et al., 2015).

In general, the neurobiology basis of mood disorders including anxiety and depression are poorly understood. A large body of studies have shown that abnormal monoamine transmitters/receptors system, altered stress hormone dynamics, deficient production of growth factors and neurotrophins, dysregulation of pro-inflammatory cytokines, disturbed adult neurogenesis, altered synaptic connectivity, oxidative stress, abnormal miRNA expression and abnormal delivery of gastrointestinal signaling peptides are all able to induce major mood alterations and associated with the pathogenesis of depression (Villanueva, 2013; Salim, 2014; Hodes et al., 2016; Boku et al., 2018). Since stress is the most common risk factor contributing to the onset and progression of depression, in this study, we applied the CSDS paradigm to *GHS-R1a* knock-out (*Ghsr*^-/-^) mice and their wild-type (*Ghsr*^+/+^) littermates which showed identical baseline anxiety and depression. We found that, CSDS facilitated social withdrawal, anxiety- and despair-like behaviors in *Ghsr*^+/+^ mice but not in *Ghsr*^-/-^ mice. To explore the underlying mechanisms, we screened the blood and brain responses of *Ghsr*^-/-^ mice and their *Ghsr*^+/+^ littermates to chronic stress, such as changes in hypothalamus-pituitary-adrenal (HPA) axis activity, pro-inflammatory cytokines expression, brain derived neurotropic factor (BDNF) expression, signaling proteins activation and oxidative stress. We found elevated BDNF in the hippocampus, and reduced IL-6 in the serum of *Ghsr*^-/-^ mice compared to *Ghsr*^+/+^ mice.

## Materials and Methods

### Animals

Male C57BL/6 mice and male CD1 mice were purchased from the Vital River Laboratory Animal Technology Co. (Beijing, China). *GHS-R1a* Knockout mice (*Ghsr*^-/-^, with *GHS-R1a* exons 1 and 2 deletion) in C57BL/6 background obtained from Shanghai Bio-model animals Research Center (Xu et al., 2012) were backcrossed >7 generations with C57BL/6 mice before expanding colony. Only adult male *Ghsr*^-/-^ mice and littermate *Ghsr*^+/+^mice with the age of 10–12 weeks old and body weight of 26–30 g were used in experiments. *Ghsr*^-/-^ mice and littermates were group-housed (two to four per cage) under a 12:12 h light/dark cycle and were given free access to water and food. CD1 mice were single-housed. The animal protocols used here were approved by the Chancellor’s Animal Research Committee at Qingdao University (in accordance with National Institutes of Health guidelines).

### Chronic Social Defeat Stress (CSDS) Paradigm

The CSDS paradigm was applied as previously described (Golden et al., 2015). Selected adult male CD1 mice with the age of 20–30 weeks old and the body weight over 40 g were housed in the defeat cages and used as aggressors in subsequent social defeat experiments. According to the previous study (Golden et al., 2015), those CD1 male mice are more aggressive. The defeat procedure was carried out between 12:00 and 16:00 pm. Over 10 days, *Ghsr*^-/-^ mice and littermate *Ghsr*^+/+^ mice were introduced into the home cage of different dominant CD1 mice for 5 min daily. All CD1 residents rapidly recognized and attacked the intruders within 2 min. After being defeated, subject *Ghsr*^-/-^ or *Ghsr*^+/+^ mice were separated from CD1 mice by a holed metal partition, allowing the subject mice continuously sense the CD1 mice without physical contact in the following 24 h after defeat. Control mice were group-housed in their home cages and allowed to explore the empty defeat cages for 5 min each day.

### Experimental Design

Behavioral tests began 1 day after defeat, which was also the 11th day of experiment. Anxiety-related behaviors were tested with a minimum inter-test interval of 8 h, all the other behavioral tests were conducted with the inter-test interval of 24 h at the least. C57BL/6J mice, *Ghsr*^-/-^ mice and littermate *Ghsr*^+/+^ mice were randomly assigned to the control or CSDS group. Sertraline ZOLOFT was purchased from Pfizer Inc. and was dissolved in DMSO to make a stock solution. Sertraline working solution (1:1,000 dilution with sterile normal saline) was freshly made and delivered intraperitoneally to a group of defeated mice (Sertraline + CSDS) at a daily dose of 20 mg/kg during both social defeat and 30 min before each behavioral test. Blood from angular vein, and brain tissues from the hippocampus were collected immediately after behavioral tests were done. Serum was isolated from fresh blood sample. Serum and brain samples were stored at -80°C freezer until analysis.

### Behavioral Tests

Behavioral tests were done between 9:00 am and 18:00 pm by the same investigator who is unaware of experimental design. Animal behaviors were video-tracked and analyzed by two independent investigators with Noldus EthoVision XT software.

An elevated plus maze (EPM) test was conducted with dim light in an elevated maze with two open and two closed arms (with walls of 16.5 cm height). Each arm is 29 cm long and 8 cm wide. Mice were released from the center and allowed to explore the maze for 5 min. Time spent in open and closed arms, number of arm entries, and total travel distance in the maze was analyzed (Cui et al., 2016; Li et al., 2018). The EPM test is a standard behavioral paradigm to evaluate anxiety-like behavior.

An open field (OF) test was often used to evaluate locomotor activity and anxiety-like behavior as well. An OF test was conducted with dim light in a square arena (28 cm × 28 cm × 35 cm) which can be divided into two zones, the center and periphery. The center was defined as a 12 cm × 12 cm zone in the middle of the open field. Each mouse released from the center was allowed to freely explore the arena for 10 min. Total distance traveled, time spend in the center or peripheral area were analyzed.

A light dark box (LDB) test was conducted in a rectangle box (40 cm × 20 cm × 25 cm) consisted of two separate compartments: a large light chamber illuminated by 60 Watt bulb, and a small dark chamber painted black and enclosed under a black cover. Mice were allowed to freely shuttle between two chambers through an opening (5 cm × 5 cm) on the wall in between. During the test, each individual mouse was gently released from the light chamber and explored the box for 10 min. Total exploration time in the light chamber was analyzed. The LDB test is also well adopted to evaluate anxiety-like behavior.

Social interaction (SI) test was widely used to test animal’s sociability. First, a testing mouse was allowed to freely explore the experimental box (30 cm × 60 cm × 25 cm) for 5 min. Then the same mouse was introduced to an unfamiliar, ovariectomized female mouse enclosed in the center of the habituated box. The testing mouse was allowed to explore the box for another 10 min. The social interaction (i.e., sniffing the female mouse within close proximity) time and frequency were recorded (Cui et al., 2016; Li et al., 2018).

During a tail suspension (TS) test, mice was gently suspended downward, tail (2 cm from the tip) being fixed with adhesive tape to a horizontal bar located 30 cm above the laboratory table. Immobility was termed as hanging passively without any voluntary movement except breath. The total immobility time and the latency to first immobility were analyzed during a 10-min hanging (Cui et al., 2016). The TS test was used to evaluate depression-like behavior.

During a forced swimming (FS) test, mice were gently released into a transparent plastic cylinder (25 cm height × 10 cm diameter) for 5 min. The cylinder was filled with water (24.5 ± 0.5°C) up to a depth of 15 cm. The water surface was 10 cm below the top of cylinder. Total immobility time and the latency to first immobility were analyzed (Cui et al., 2016). Similar to TS test, FS test was often used to evaluate depression-like behavior.

### RNA Extraction and qRT-PCR

Total RNA (1 μg) was extracted from the hippocampus using a PureLink^TM^ RNA Mini Kit (Thermo Fisher Scientific) and reverse-transcribed into cDNA with a SuperScript^TM^ III First-Strand Synthesis SuperMix (Invitrogen) according to the manufacturer’s instruction. RNA quantity and quality was determined with a NanoDrop 2000 Spectrophotometer (Thermo Fisher Scientific). qPCR-based quantification of *Ghsr* was performed using a MasterCycler ep realplex PCR system (Eppendorf) and a QuantiFast SYBR Green PCR Kit (Qiagen). The PCR cycling parameters were as follows: 95°C for 5 min, followed by 40 cycles of PCR reaction at 95°C for 5 s, 60°C for 30 s, 72°C for 30 s. *Actb* was used as the housekeeping control for all samples. The expression of *Ghsr* in the *Ghsr*^-/-^ mice was normalized to that observed in the *Ghsr*^+/+^ mice. PCR primer sequences used were as follows: *Ghsr*-F *GTATGGGTGTCGAGC GTCTT*, *Ghsr*-R *AGCCAGCAGAGGATGAAAGC*; *Actb*-F*CATCCGTAAAGACCTCTATGC CAAC*, *Actb*-F *ATGGAGCCACCGATCCACA*.

### Blood Serum Isolation and Brain Tissue Preparation

About 200 μl of whole blood was freshly taken from each mouse and kept in a sterile PE tube without any anticoagulant. Blood sample was left at room temperature for 30 min to form a clot. Immediately after centrifuging at 1,500 × *g* for 10 min at 4°C, the resulting supernatant serum was quickly transferred into a clean PE tube using a Pasteur pipette. Samples were maintained at 4°C while handling and stored at -80°C until use.

The hippocampus and the PFC were isolated on ice according to the manufacturer’s instruction. The hippocampus tissue was homogenized in 0.5 ml ELISA buffer, sonicated for 5 s and centrifuged at 3,500 × *g* for 10 min to collect the resulting supernatant. The PFC tissue were lysed in RIPA lysis buffer and centrifuged at 3,000 × *g* for 15 min to obtain the resulting supernatant. All brain samples were handled at 4°C and the supernatants were quickly stored at -80°C until use.

### Enzyme-Linked Immunosorbent Assay (ELISA)

The concentration of BDNF in the hippocampus, the concentration of total ghrelin, leptin, ACTH, and corticosterone in both blood serum and the hippocampus, and the serum concentration of interleukin-1 beta (IL-1β), interleukin-6 (IL-6), interleukin-18 (IL-18), tumor necrosis factor-alpha (TNF-α) and interferon-gamma (IFN-γ) were measured with corresponding mouse ELISA kits (Wuhan Colorful Gene Biological Technology Co., China) according to the manufacturer’s instruction. Absorbance value for each sample running in triplicate was measured at 450 nm using a 96-well microplate spectrophotometer. Protein concentration was then calculated from the standard curve plotted by absorbance values of a diluted series of standards provided in each kit.

Concentrations of IL-1β, IL-6, IL-18, TNF-α, and IFN-γ in hippocampal tissue were simultaneously quantified using a MILLIPLEX^®^MAP mouse cytokine/chemokine magnetic bead panel (MCYTOMAG-70K-05 mouse, Merck-Millipore). Protein concentration was measured, adjusted to 1 mg/ml and diluted 1:1 with assay buffer. A total of 25 μl sample was introduced into a plate prepared following the manufacturer’s protocol. Mean fluorescent intensities were determined by MAGPIX^®^with xPONENT^®^software (Luminex^®^Co.). Cytokine concentrations were analyzed in the Merck-Millipore lab in China.

Specific enzyme activity assay kits for superoxide dismutase (SOD), catalase (CAT) and malondialdehyde (MDA) were purchased from Nanjing Jiancheng Bioengineering Institute (Nanjing, China). SOD, CAT, and MDA activity in the PFC sample were measured at 550, 405, and 532 nm, respectively, by spectrophotometer according to the manufacturer’s instruction.

### Multiplex Assay of Cell Signaling Pathways

Activity of AKT, CREB, ERK, JNK, and p38MAPK in hippocampal tissue were simultaneously measured using a MILLIPLEX^®^MAP Cell Signaling 2-Plex Assay kit (MAPmate phosphor- and total-protein Plex-6 assay, Merck-Millipore). Mean fluorescent intensities were determined by MAGPIX^®^with xPONENT^®^software (Luminex^®^Co.). Phosphorylation levels and total levels of Akt/PBK (ser473), CREB (Ser133), ERK/MAPK1/2 (thr185/tyr187), JNK/SAPK1 (thr183/tyr185), and p38MAPK/SAPK2 (thr180/Tyr182) were analyzed in the Merck-Millipore lab in China. Phosphorylated protein/total protein ratio represents kinase activity.

### Statistical Analyses

Data were expressed as mean ± S.E.M. Statistical analyses were performed with GraphPad Prism 6.0 software. ANOVAs or *t*-tests were used for statistical comparisons between groups as described in the main context. The significance level was set to *P* < 0.05.

## Results

### *Ghsr*^-/-^ Mice Exhibited Normal Anxiety- and Depression-Like Behaviors at Baseline State

**FIGURE 1:**
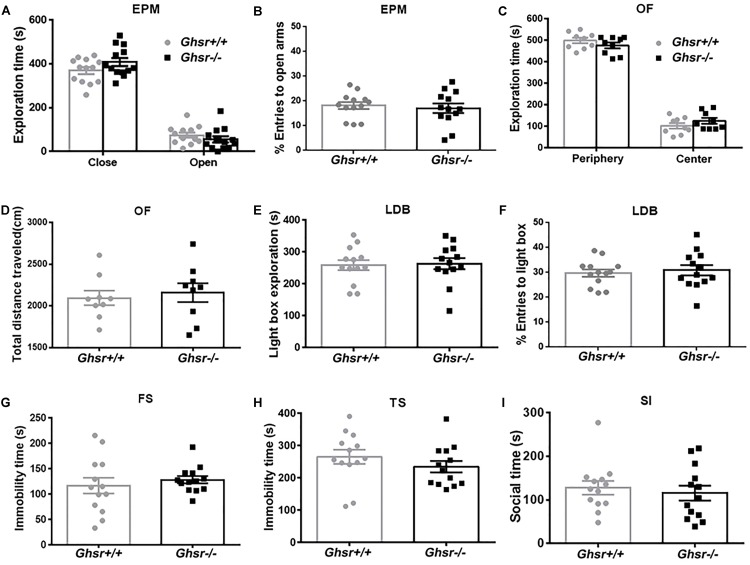
*Ghsr*^-/-^ mice exhibited normal anxiety- and depression-like behaviors at baseline state. **(A,B)** EPM test in *Ghsr*^-/-^ mice and control *Ghsr*^+/+^ mice. **(A)** Time exploring open or close arms. **(B)** The percentage of open arms entries. Two-way repeated-measure ANOVA in **(A)** and unpaired *t*-test in **(B)**, *n* = 13 mice for each group. **(C,D)** OF test in *Ghsr*^-/-^ mice and *Ghsr*^+/+^ littermates. **(C)** Time exploring the center or peripheral arena in the OF test. **(D)** Total distance traveled in the open field. Two-way repeated-measure ANOVA in **(C)** and unpaired *t*-test in **(D)**, *n* = 9 for each group. **(E,F)** LBD test. **(E)** Time exploring the light box. **(F)** The percentage of light box entries. **(G)** Immobility in the FS test. **(H)** Immobility in TS test. **(I)** Sociability in social SI test. In **(E–I)**, unpaired *t*-test, *n* = 13 for each group. All data are shown as means ± SEM.

At baseline state, *Ghsr*^-/-^ mice and *Ghsr*^+/+^ littermates spent similar time exploring open or close arms in an EPM test [Figure 1A; two-way repeated-measure ANOVA with between-subjects factors genotype and arms, genotype *F*_(1,24)_ = 2.7, *P* > 0.05; genotype × arms *F*_(1,24)_ = 2.1, *P* > 0.05; arms *F*_(1,24)_ = 255.0, *P* < 0.0001; Sidak’s multiple comparisons test, *P* > 0.05 for either arms comparison], and the percentage of open arms entries were also similar between two groups of mice (Figure 1B; unpaired *t*-test, *t* = 0.5, *P* > 0.05). *Ghsr*^-/-^ mice and *Ghsr*^+/+^ littermates spent similar time exploring center or peripheral arena in an OF test [Figure 1C; two-way repeated-measure ANOVA with between-subjects factors genotype and arena, genotype *F*_(1,16)_ = 2.3, *P* > 0.05; genotype × arena *F*_(1,16)_ = 1.6, *P* > 0.05; arena *F*_(1,16)_ = 401.3, *P* < 0.0001; Sidak’s multiple comparisons test, *P* > 0.05 for either arena comparison], and the total distance traveled in OF were also comparable between two groups of mice (Figure 1D; unpaired *t*-test, *t* = 0.4, *P* > 0.05). In addition, *Ghsr*^-/-^ mice and *Ghsr*^+/+^ littermates spent similar time exploring light box in a LBD test (Figure 1E; unpaired *t*-test, *t* = 0.2, *P* > 0.05). The percentage of light box entries were similar as well between two groups of mice (Figure 1F; unpaired *t*-test, *t* = 0.4, *P* > 0.05). All these results indicated that *Ghsr*^-/-^ mice had normal locomotor activity, same baseline anxiety as *Ghsr*^+/+^ mice. Moreover, these two groups mice showed similar despair-like behaviors at baseline state, as their immobility time in both the FS test (Figure 1G; unpaired *t*-test, *t* = 0.7, *P* > 0.05) and the TS test (Figure 1H; unpaired *t*-test, *t* = 1.1, *P* > 0.05) were comparable. The SI test indicated normal sociability of *Ghsr*^-/-^ mice, as they exhibited same social interaction time as *Ghsr*^+/+^ mice (Figure 1I; unpaired *t*-test, *t* = 0.5, *P* > 0.05). Therefore, all these findings demonstrated that GHS-R1a deficiency did not affect anxiety- and depression-like behaviors at baseline state without chronic stress exposure.

### *Ghsr*^-/-^ Mice Exhibited Enhanced Behavioral Resistance to Anxiety and Depression After CSDS Than *Ghsr*^+/+^ Mice

Chronic social defeat stress is a well-accepted animal model for depression. Previous studies reported that mice subjected to CSDS exhibited lasting behavioral deficits, including social avoidance, depression and anxiety (Hollis and Kabbaj, 2014; Golden et al., 2015), as well as significant elevation in circulating ghrelin (Lutter et al., 2008). We first validated this model in wild-type mice (Supplementary Figure S1A). Indeed, we confirmed that C57BL/6 male mice displayed severe emotion deficit, such as increased anxiety- and despair-like behaviors, after repeated social defeat for 10 days by aggressive CD1 mice (data not shown). Meanwhile, we observed significant increase in serum concentration of ACTH (Supplementary Figure S1B; unpaired *t*-test, *t* = 2.9, *P* < 0.05), TNF-α (Supplementary Figure S1C; unpaired *t*-test with Welch’s correction, *t* = 2.6, *P* < 0.05) and IL-6 (Supplementary Figure S1D; unpaired *t*-test with Welch’s correction, *t* = 3.7, *P* < 0.01) in defeated mice compared to home-cage control mice, supporting the hyperactivity of HPA axis and pro-inflammation in response to chronic social stress (Hollis and Kabbaj, 2014; Spencer et al., 2015; Takahashi et al., 2018). We also assessed *Ghsr* expression by qPCR and confirmed dramatically reduced expression in the hippocampus of *Ghsr^-^*^/^*^-^* mice compared to wild-type *Ghsr*^+/+^ littermates (Supplementary Figure S1E; unpaired *t-*test with Welch’s correction, *t* = 4.7, *P* < 0.01).

**FIGURE 2:**
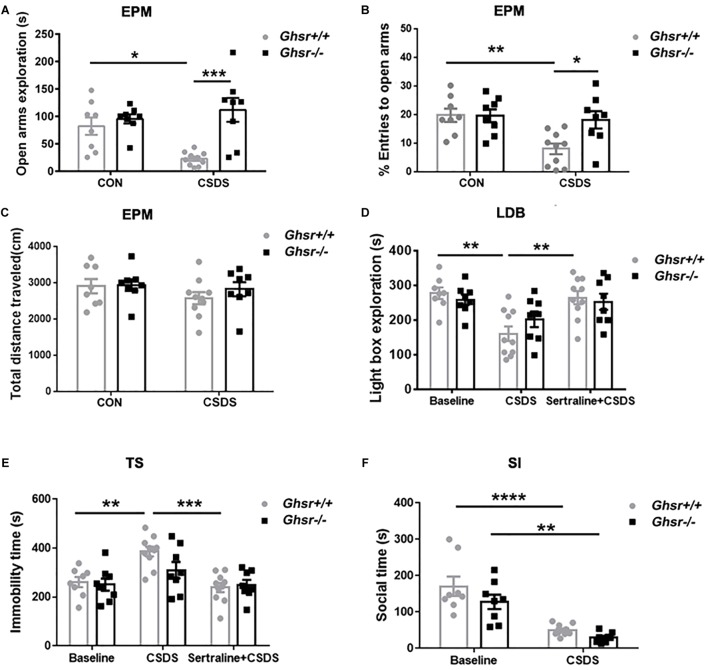
*Ghsr*^-/-^ mice exhibited more resistance to CSDS than *Ghsr*^+/+^ mice. **(A–C)** EPM test both at baseline state (CON) and after CSDS. **(A)** *Ghsr*^+/+^ mice showed reduced open-arm exploration and **(B)** reduced percentage of open arms entries after CSDS, while *Ghsr^-^*^/^*^-^* mice did not. **(C)** Total distance traveled in the maze were similar between *Ghsr^-^*^/^*^-^* mice and control *Ghsr*^+/+^ mice. *Ghsr*^+/+^ mice, *n* = 8 for control group and *n* = 10 for CSDS group. *Ghsr*^-/-^ mice, *n* = 8 for each group. **(D)** LDB test. *Ghsr*^+/+^ mice showed less light-box exploration after CSDS, which could be rescued by sertraline administration. *Ghsr*^+/+^ mice, *n* = 8 for control group, *n* = 10 for CSDS group and *n* = 10 for Sertraline + CSDS group. *Ghsr*^-/-^ mice, *n* = 8 for control group, *n* = 8 for CSDS group and *n* = 8 for Sertraline + CSDS group. **(E)** TS test. *Ghsr*^+/+^ mice showed increased immobility after CSDS, which could be rescued by sertraline administration. *Ghsr*^+/+^ mice, *n* = 8 for control group, *n* = 10 for CSDS group and *n* = 10 for sertraline + CSDS group. *Ghsr*^-/-^ mice, *n* = 8 for each group. **(F)** SI test. Both *Ghsr*^+/+^ mice and *Ghsr*^-/-^ mice showed comparable, significantly reduced social interaction after CSDS. *Ghsr*^+/+^ mice, *n* = 8 for control group and *n* = 10 for CSDS group; *Ghsr*^-/-^ mice, *n* = 8 for each group. Two-way ANOVA with between-subjects factors genotype and treatment. ^∗^*P* < 0.05, ^∗∗^*P* < 0.01, ^∗∗∗^*P* < 0.001, *^∗∗∗∗^P* < 0.0001 means significant difference. All data are shown as means ± SEM.

We then applied CSDS procedure to *Ghsr^-^*^/^*^-^* mice and *Ghsr*^+/+^ littermates in order to determine whether ghrelin/GHS-R1a signaling regulates stress response, anxiety- and depression-like behaviors under CSDS, a chronic psychological stress state. We found that, *Ghsr*^+/+^ mice exhibited significant anxiety- and depression-like behaviors after chronic social defeat while *Ghsr*^-/-^ mice did not. As shown in Figure 2, defeated *Ghsr*^+/+^ mice showed less open-arm exploration [Figure 2A; two-way ANOVA with between-subjects factors genotype and treatment, genotype *F*_(1,30)_ = 14.3, *P* < 0.001; genotype × treatment *F*_(1,30)_ = 7.9, *P* < 0.01; treatment *F*_(1,30)_ = 2.5, *P* > 0.05; Tukey’s multiple comparisons test, *Ghsr*^+/+^ mice CSDS vs. CON, *P* < 0.05] and less open-arm entries [Figure 2B; two-way ANOVA with between-subjects factors genotype and treatment, genotype *F*_(1,30)_ = 4.4, *P* < 0.05; genotype × treatment *F*_(1,30)_ = 4.7, *P* < 0.05; treatment *F*_(1,30)_ = 7.6, *P* < 0.01; Tukey’s multiple comparisons test, *Ghsr*^+/+^ mice CSDS vs. CON, *P* < 0.01] in a EPM test, however, the total distance traveled in four arms were comparable among four groups of mice (Figure 2C; two-way ANOVA with between-subjects factors genotype and treatment, *P* > 0.05) indicating that CSDS did not affect locomotor activity of either *Ghsr*^+/+^ or *Ghsr*^-/-^ mice. Defeated *Ghsr*^+/+^ mice also displayed less light-box exploration in a LDB test [Figure 2D; two-way ANOVA with between-subjects factors genotype and treatment, treatment *F*_(2,46)_ = 11.5, *P* < 0.0001; genotype *F*_(1,46)_ = 0.1, *P* > 0.05; genotype × treatment *F*_(2,46)_ = 1.4, *P* > 0.05; Tukey’s multiple comparisons test, *Ghsr*^+/+^ mice CSDS vs. CON, *P* < 0.01], and increased immobility in a TS test [Figure 2E; two-way ANOVA with between-subjects factors genotype and treatment, treatment *F*_(2,46)_ = 12.2, *P* < 0.0001; genotype *F*_(1,46)_ = 1.8, *P* > 0.05; genotype × treatment *F*_(2,46)_ = 1.9, *P* > 0.05; Tukey’s multiple comparisons test, *Ghsr*^+/+^ mice CSDS vs. CON, *P* < 0.01], in comparison to non-stressed control *Ghsr*^+/+^ mice. Moreover, intraperitoneal injection of sertraline, a selective serotonin reuptake inhibitor (SSRI) antidepressant during CSDS procedure, prevent the development of increased anxiety-like behavior (Figure 2D; *Ghsr*^+/+^ mice CSDS vs. Sertraline + CSDS, *P* < 0.01) and despair-like behavior (Figure 2E; *Ghsr*^+/+^ mice CSDS vs. Sertraline + CSDS, *P* < 0.001) induced by CSDS exposure.

However, distinct from what we saw in *Ghsr*^+/+^ mice, *Ghsr*^-/-^ mice exposed to CSDS displayed similar open-arm exploration time and open-arm entries as non-stressed *Ghsr*^-/-^ mice in a EPM test (Figure 2A,B; *Ghsr*^-/-^ mice CSDS vs. CON, *P* > 0.05). *Ghsr*^-/-^ mice exposed to CSDS also showed comparable light-box exploration in a LDB test (Figure 2D; *Ghsr*^-/-^ mice CSDS vs. CON, *P* > 0.05), and comparable immobility in a TS test (Figure 2E; *Ghsr*^-/-^ mice CSDS vs. CON, *P* > 0.05) to non-stressed *Ghsr*^-/-^ mice. Also, intraperitoneal injection of sertraline during the CSDS procedure did not affect anxiety-like behavior (Figure 2D; *Ghsr*^-/-^ mice CSDS vs. Sertraline + CSDS, *P* > 0.05) or despair-like behavior (Figure 2E; *Ghsr*^-/-^ mice CSDS vs. Sertraline + CSDS, *P* > 0.05) of *Ghsr*^-/-^ mice. Those findings suggested that *Ghsr*^-/-^ mice may be resistant or invulnerable to CSDS-induced affective deficit, such as anxiety and despair. Interestingly, we did not find difference between *Ghsr*^+/+^ mice and *Ghsr*^-/-^ mice as for sociability changes after CSDS. Both *Ghsr*^+/+^ mice and *Ghsr*^-/-^ mice showed comparable, significantly reduced social interaction after defeat [Figure 2F; two-way ANOVA with between-subjects factors genotype and treatment, treatment *F*_(1,30)_ = 46.4, *P* < 0.0001; genotype *F*_(1,30)_ = 3.9, *P* > 0.05; genotype × treatment *F*_(1,30)_ = 0.5, *P* > 0.05; Tukey’s multiple comparisons test, *Ghsr*^+/+^ mice CSDS vs. CON, *P* < 0.0001; *Ghsr*^-/-^ mice CSDS vs. CON, *P* < 0.01; CSDS *Ghsr*^+/+^ mice vs. CSDS *Ghsr*^-/-^ mice, *P* > 0.05]. Notably, *Ghsr*^+/+^ mice and *Ghsr*^-/-^ mice exhibited same behavioral performance at the control, non-stressed state (Figure 2A–F, *p* > 0.05). Altogether, our data demonstrated that *Ghsr*^-/-^ mice showed enhanced behavioral resistance to CSDS-induced mood disorders than *Ghsr*^+/+^ mice, suggesting beneficial effect of *Ghsr* deficiency on mood regulation, in particular anxiety.

### *Ghsr*^+/+^ Mice and *Ghsr*^-/-^ Mice Showed Different Blood and Brain Responses to CSDS

To explore the possible mechanism mediating the beneficial effect of *Ghsr* deficiency on mood regulation after CSDS exposure, we checked and compared the peripheral and central responses of *Ghsr*^-/-^ mice and *Ghsr*^+/+^ littermates to chronic stress, including ghrelin and leptin levels, HPA axis activity, pro-inflammatory cytokines concentration in both serum and the hippocampus, BDNF expression and cell signaling pathways activation in the hippocampus and oxidative stress in the PFC. First of all, we checked multiple peripheral and central biomarkers at baseline, non-stressed state and found no difference between *Ghsr*^-/-^ mice and *Ghsr*^+/+^ littermates (Supplementary Figure S2, unpaired *t*-test, *P* > 0.05).

**FIGURE 3:**
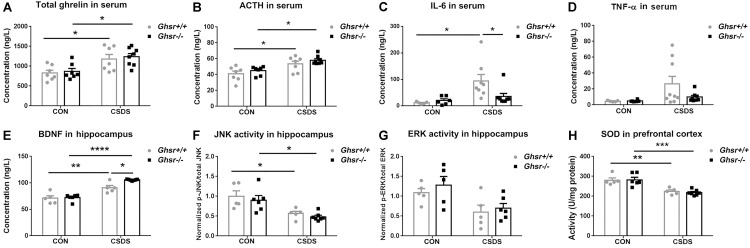
*Ghsr*^+/+^ mice and *Ghsr*^-/-^ mice showed different peripheral and central responses to CSDS. **(A)** Total ghrelin and **(B)** ACTH in serum of *Ghsr*^+/+^ mice and *Ghsr*^-/-^ mice, measured both at the baseline state (CON) and after CSDS. *Ghsr*^+/+^ CON, *n* = 7; *Ghsr*^-/-^ CON, *n* = 7; *Ghsr*^+/+^ CSDS, *n* = 7; *Ghsr*^-/-^ CSDS, *n* = 8. **(C)** IL-6 in serum. *Ghsr*^+/+^ CON, *n* = 5; *Ghsr*^-/-^ CON, *n* = 6; *Ghsr*^+/+^ CSDS, *n* = 8; *Ghsr*^-/-^ CSDS, *n* = 8. **(D)** TNF-α in serum. *Ghsr*^+/+^ CON, *n* = 5; *Ghsr*^-/-^ CON, *n* = 6; *Ghsr*^+/+^ CSDS, *n* = 9; *Ghsr*^-/-^ CSDS, *n* = 8. **(E)** BDNF and **(F)** JNK activity in the hippocampus. *Ghsr*^+/+^ CON, *n* = 5; *Ghsr*^-/-^ CON, *n* = 6; *Ghsr*^+/+^ CSDS, *n* = 5; *Ghsr*^-/-^ CSDS, *n* = 7. **(G)** ERK activity in the hippocampus. *Ghsr*^+/+^ CON, *n* = 5; *Ghsr*^-/-^ CON, *n* = 5; *Ghsr*^+/+^ CSDS, *n* = 5; *Ghsr*^-/-^ CSDS, *n* = 6. **(H)** SOD activity in prefrontal cortex. *Ghsr*^+/+^ CON, *n* = 5; *Ghsr*^-/-^ CON, *n* = 6; *Ghsr*^+/+^ CSDS, *n* = 5; *Ghsr*^-/-^ CSDS, *n* = 7. Two-way ANOVA with between-subjects factors genotype and treatment. ^∗^*P* < 0.05, ^∗∗^*P* < 0.01, ^∗∗∗^*P* < 0.001, *^∗∗∗∗^P* < 0.0001 means significant difference. All data are shown as means ± SEM.

Consistent with previous reports (Lutter et al., 2008; Meyer et al., 2014; Huang et al., 2017), we found that CSDS elevated ghrelin [Figure 3A; two-way ANOVA with between-subjects factors genotype and treatment, treatment *F*_(1,25)_ = 17.1, *P* < 0.001; Tukey’s multiple comparisons test, *Ghsr*^-/-^ CSDS vs. *Ghsr*^-/-^ CON, *P* < 0.05; *Ghsr*^+/+^ CSDS vs. *Ghsr*^+/+^ CON, *P* < 0.05] and ACTH [Figure 3B; two-way ANOVA with between-subjects factors genotype and treatment, treatment *F*_(1,25)_ = 20.8, *P* = 0.0001; Tukey’s multiple comparisons test, *Ghsr*^-/-^ CSDS vs. *Ghsr*^-/-^ CON, *P* < 0.05; *Ghsr*^+/+^ CSDS vs. *Ghsr*^+/+^ CON, *P* < 0.05] in the serum of both *Ghsr*^-/-^ mice and *Ghsr*^+/+^ mice. *Ghsr*^-/-^ mice and *Ghsr*^+/+^ mice showed equivalent levels of serum ghrelin and ACTH either at baseline state or after CSDS, indicating no genotype or interaction effect [Figure 3A,B; genotype *F*_(1,25)_ = 0.3 or 2.5, *P* > 0.05; genotype × treatment *F*_(1,25)_ = 0.01, *P* > 0.05; Tukey’s multiple comparisons test, *Ghsr*^-/-^ mice vs. *Ghsr*^+/+^ mice, *P* > 0.05]. In addition, there was no difference between *Ghsr*^-/-^ mice and *Ghsr*^+/+^ mice regarding serum concentrations of leptin and corticosterone, both at baseline state and after CSDS (data not shown), suggesting normal HPA axis activity of *Ghsr*^-/-^ mice responding to CSDS.

Since increased levels of pro-inflammatory cytokines is associated with social stress and the pathogenesis of anxiety and depression (Mellon et al., 2018; Takahashi et al., 2018), we checked alterations of pro-inflammatory cytokines after CSDS exposure in *Ghsr*^-/-^ mice and *Ghsr*^+/+^ littermates. Interestingly, we found that CSDS elevated IL-6 level in serum of *Ghsr*^+/+^ mice [Figure 3C; two-way ANOVA with between-subjects factors genotype and treatment, treatment *F*_(1,23)_ = 8.6, *P* < 0.01; genotype *F*_(1,23)_ = 2.3, *P* > 0.05; genotype × treatment *F*_(1,23)_ = 4.2, *P* = 0.05; Tukey’s multiple comparisons test, *Ghsr*^+/+^ mice CSDS vs. CON, *P* < 0.05], but not in *Ghsr*^-/-^ mice (Figure 3C; *Ghsr*^-/-^ mice CSDS vs. CON, *P* > 0.05). The serum concentration of IL-6 in defeated *Ghsr*^-/-^ mice was lower than in defeated *Ghsr*^+/+^ mice (Figure 3C; *Ghsr*^-/-^ CSDS vs. *Ghsr*^+/+^ CSDS, *P* < 0.05). Similarly, we also noticed that CSDS tended to increase serum TNF-α level specifically in defeated *Ghsr*^+/+^ mice but not in defeated *Ghsr*^-/-^ mice [Figure 3D; two-way ANOVA with between-subjects factors genotype and treatment, treatment *F*_(1,24)_ = 4.2, *P* = 0.05]. There was no difference between defeated *Ghsr*^-/-^ mice and defeated *Ghsr*^+/+^ mice regarding serum concentrations of other pro-inflammation cytokines including IL-1β and IFN-γ (data not shown). Our results thus suggested less pro-inflammatory activity in defeated *Ghsr*^-/-^ mice than in defeated *Ghsr*^+/+^ mice.

Since BDNF has emerged as a possible biomarker for depression (Deng et al., 2010; Eisch and Petrik, 2012), we compared BDNF concentration in the hippocampus of *Ghsr*^-/-^ mice and *Ghsr*^+/+^ littermates, at baseline state and after CSDS exposure. We found that CSDS elevated BDNF concentration in the hippocampus of both *Ghsr*^-/-^ mice and *Ghsr*^+/+^ mice [Figure 3E; two-way ANOVA with between-subjects factors genotype and treatment, treatment *F*_(1,19)_ = 77.1, *P* < 0.0001; genotype *F*_(1,19)_ = 7.1, *P* < 0.05; genotype × treatment *F*_(1,19)_ = 5.0, *P* < 0.05; Tukey’s multiple comparisons test, *Ghsr*^-/-^ CSDS vs. CON, *P* < 0.0001; *Ghsr*^+/+^ CSDS vs. CON, *P* < 0.01]. Interestingly, *Ghsr*^-/-^ mice exhibited higher level of BDNF in the hippocampus than *Ghsr*^-/-^ mice after CSDS exposure, while their BDNF concentrations at baseline state were same (Figure 3E; *Ghsr*^-/-^ CSDS vs. *Ghsr*^+/+^ CSDS, *P* < 0.05; *Ghsr*^-/-^ CON vs. *Ghsr*^+/+^ CON, *P* > 0.05).

Ghrelin/GHS-R1a was reported to engage in multiple cell signaling pathways participating in the neuronal modulation of stress response and depression (Duman and Voleti, 2012), which include ERK1/2, p38MAPK, c-Jun-*N*-terminal kinase/stress-activated protein kinase (JNK/SAPK), Akt, CREB, and etc. We then checked the activities of those signaling pathways in the hippocampus of *Ghsr*^+/+^ mice and *Ghsr*^-/-^ mice, both at baseline state and after CSDS exposure. We found that CSDS suppressed JNK activity in the hippocampus of both *Ghsr*^+/+^ mice and *Ghsr*^-/-^ mice [Figure 3F; two-way ANOVA with between-subjects factors genotype and treatment, treatment *F*_(1,19)_ = 20.6, *P* < 0.001; genotype *F*_(1,19)_ = 0.9, *P* > 0.05; genotype × treatment *F*_(1,19)_ = 0.01, *P* > 0.05; Tukey’s multiple comparisons test, *Ghsr*^-/-^ CSDS vs. CON, *P* < 0.05; *Ghsr*^+/+^ CSDS vs. CON, *P* < 0.05]. However, there was no difference between *Ghsr*^+/+^ mice and *Ghsr*^-/-^ mice regarding hippocampal JNK activity either at baseline state or after CSDS (Figure 3F; *Ghsr*^-/-^ CSDS vs. *Ghsr*^+/+^ CSDS, *P* > 0.05; *Ghsr*^-/-^ CON vs. *Ghsr*^+/+^ CON, *P* > 0.05). Meanwhile, ERK1/2 activity in the hippocampus also tended to be suppressed by CSDS in our study [Figure 3G; two-way ANOVA with between-subjects factors genotype and treatment, treatment *F*_(1,17)_ = 10.9, *P* < 0.01; genotype *F*_(1,19)_ = 0.9, *P* > 0.05; genotype × treatment *F*_(1,19)_ = 0.1, *P* > 0.05], which was consistent with previous reports (Lio et al., 2011). Similarly, we did not found any difference between *Ghsr*^+/+^ mice and *Ghsr*^-/-^ mice regarding hippocampal ERK activity either at baseline state or after CSDS (Figure 3G; *Ghsr*^-/-^ CSDS vs. *Ghsr*^+/+^ CSDS, *P* > 0.05; *Ghsr*^-/-^ CON vs. *Ghsr*^+/+^ CON, *P* > 0.05). In addition, we did not find treatment (CSDS vs. CON) or genotype (*Ghsr*^+/+^ vs. *Ghsr*^-/-^) effect on other signaling pathway activities in *Ghsr*^-/-^ mice and *Ghsr*^+/+^ mice, including Akt, CREB, and p38 MAPK (data not shown).

Oxidative stress was also reported to be implicated in several mental disorders including depression and anxiety (Salim, 2014), therefore we checked SOD and CAT activities in the PFC of *Ghsr*^+/+^ mice and *Ghsr*^-/-^ mice, both at baseline state and after CSDS. We found decreased SOD activity in both defeated *Ghsr*^+/+^ mice and defeated *Ghsr*^-/-^ mice indicating the existence of persisting central oxidative stress after CSDS exposure [Figure 3H; two-way ANOVA with between-subjects factors genotype and treatment, treatment *F*_(1,19)_ = 41.0, *P* < 0.0001; genotype *F*_(1,19)_ = 0.1, *P* > 0.05; genotype × treatment *F*_(1,19)_ = 0.2, *P* > 0.05; Tukey’s multiple comparisons test, *Ghsr*^-/-^ CSDS vs. CON, *P* < 0.001; *Ghsr*^+/+^ CSDS vs. CON, *P* < 0.01]. However, *Ghsr*^+/+^ mice and *Ghsr*^-/-^ mice showed no difference regarding SOD activity either at baseline state or after CSDS, suggesting comparable central antioxidant responses to chronic stress in those two groups mice (Figure 3H; *Ghsr*^-/-^ CSDS vs. *Ghsr*^+/+^ CSDS, *P* > 0.05; *Ghsr*^-/-^ CON vs. *Ghsr*^+/+^ CON, *P* > 0.05). We did not observe any treatment or genotype effect on CAT activity in the hippocampus of *Ghsr*^-/-^ mice or control *Ghsr*^+/+^ mice (data not shown). Altogether, our data demonstrated that *Ghsr*^-/-^ mice displayed different peripheral and central responses to CSDS compared to *Ghsr*^+/+^ mice, with higher level of BDNF in the hippocampus and lower level of IL-6 in the serum, which may correlate to improved behavioral resistance to CSDS.

## Discussion

Stress responses are physiological responses to life-threatening cues or events, a survival mechanism engaged to promote coping and adaptation. However, repeated or prolonged activation of the stress response causes detrimental effects or maladaptation, including increased susceptibility to post-traumatic stress disorder (PTSD), depression and anxiety (McEwen, 1998, 2003). Hormones in the HPA axis are believed to mediate the body’s response to an acute or a chronic physical or psychological stressor (Sapolsky et al., 2000). Studies suggested that ghrelin is a crucial element regulating the HPA axis therefore contributes to the development of stress-related mood disorders, including anxiety, depression and fear (Spencer et al., 2015). Interestingly, a recent finding uncovered an essential role of ghrelin-growth hormone axis in the amygdala, which acted in parallel to the classic HPA stress axis, to drive chronic stress-induced susceptibility to enhanced fear, a key feature of PTSD (Meyer et al., 2014). The same group further reported that chronic, but not acute restraint stress, increases circulating acyl-ghrelin meanwhile promotes central ghrelin resistance in the amygdala, which permits enhanced fear memory formation (Harmatz et al., 2017). In contrast to these findings showing that ghrelin mediates maladaptive changes following prolonged stress, other studies have argued that ghrelin promotes adaptive changes during stress, including antidepressant effects (Lutter et al., 2008; Huang et al., 2017) and reduce anxiety (Spencer et al., 2012; Mahbod et al., 2018). Limited human studies to date reported variable associations, either positive, negative, or not existing, between ghrelin and mood disorders. Therefore, although a variety of chronic stressors, such as repeated tail pinch, daily restraint, chronic unpredictable stress and CSDS in rats and mice, cause persistent elevation in plasma ghrelin, the resulting potentiation of ghrelin/GHS-R1a system seems to play distinct roles, either maladaptive or adaptive, in the development of increased vulnerability to anxiety and depression after chronic stress exposure. The reason for such disparate effect are unknown yet.

In this study, we compared the anxiety- and depression-like behaviors in GHS-R1a knock-out mice and their wild-type littermates both at baseline, non-stress state and after CSDS. We found that *Ghsr* knock-out did not affect mice baseline behaviors, the two groups mice exhibited identical locomotor activity, sociability, anxiety and etc., which is consistent with previous reports (Lutter et al., 2008; Spencer et al., 2012; Mahbod et al., 2018). However, their behavioral performance after CSDS exposure was distinct. Stressed wild-type control mice showed dramatic social withdrawal, anxiety- and despair-like behaviors; while stressed knock-out mice only showed sociability deficit. Our findings thus suggested that GHS-R1a deficiency could alleviate depressive symptoms of chronic stress, that is to say, chronic stress-induced endogenous ghrelin/GHS-R1a signaling may promote anxiety- and depression-like behaviors. Our results are supported by a previous study showing that central administration of ghrelin for 4 weeks increases anxiety- and depression-like behaviors in rats (Hansson et al., 2011), whereas are in conflict with another earlier study showing that ghrelin/GHS-R1a system plays antidepressant effects against CSDS (Lutter et al., 2008). It is worth mentioned that, in the latter study, the antidepressant effect of *Ghsr* knock-out was only a mild improvement of a stress-related impairment in social interaction. It is also important to note that, in the latter study, elevated ghrelin levels were achieved by extreme food deprivation or a single bolus injection, which may have profoundly different effect from repeated or prolonged ghrelin manipulations. Also, food deprivation drives food-seeking behaviors and increases exploratory activity which may confound measurement of social interaction. In particular, one piece of data showed that patients with treatment-resistant major depressive disorder have higher ghrelin levels than control patients (Ishitobi et al., 2012).

Immobility in FST and TST is commonly interpreted as depression-like state, however, recent studies demonstrated that increased floating/immobility during repeated FST may rather reflect an adaptive, learned behavioral response underlying survival than depression (de Kloet and Molendijk, 2016). To be mentioned, in our experiment, FST and TST were only carried out once, but not repeatedly. Also, Mul et al. (2016, eNeuro) stated in their study that the initial assessment of depression-like behavior in rodents can include treatment-based screens, such as the FST and TST. Therefore, we still adopted immobility behavior in FST and TST as one of the parameters of depression. However, a combination of emotional symptoms (anhedonia), homeostatic symptoms (sleep, appetite, and body weight), and psychomotor symptoms (locomotor activity, immobility, and anxiety-like behavior) should be measured in the future to provide more definitive evidence of a depression-like state (Nestler and Hyman, 2010).

The neurobiology basis underlying depression has not been fully identified. Basic and clinical studies demonstrated that chronic stress and depression are associated with decreased size and function of limbic brain regions, including the PFC, hippocampus and amygdala (Mayberg, 2009; Duman and Voleti, 2012). BDNF facilitates neurogenesis and neuroplasticity in the hippocampus, therefore may be critical for mood regulation (Deng et al., 2010; Eisch and Petrik, 2012). Indeed, BDNF was reported to be sufficient to produce an antidepressant response in behavioral models of depression (Duman and Monteggia, 2006; Schmidt and Duman, 2007). We compared BDNF concentration in the hippocampus of *Ghsr*^-/-^ mice and *Ghsr*^+/+^ controls, both at baseline state and after CSDS exposure. To be surprised, we found that BDNF level in the hippocampus was elevated after CSDS, which is inconsistent with previous studies showing that stress decreases and antidepressant treatment increases the expression of BDNF in the hippocampus and PFC (Duman and Monteggia, 2006; Krishnan and Nestler, 2008; Castren and Rantamaki, 2010). A very recent study also reported that CSDS failed to induce any changes in total *bdnf* gene expression in the hippocampus of social defeated C57BL6 mice (Martin et al., 2017). The discrepancy may be due to different stress state of the tested animal, for example, acute vs. chronic stress, mild vs. strong stress. It is also possible that elevated BDNF in the hippocampus is certain homeostatic compensation/plasticity adapted to chronic stress. Since *Ghsr*^-/-^ mice showed normal baseline level of BDNF, but higher concentration of BDNF after CSDS than *Ghsr*^+/+^ control mice, we proposed that such elevation may associate with GHS-R1a deficiency-induced behavioral resistance to CSDS, such as reduced anxiety and despair. Further studies, especially study based on conditional GHS-R1a knockout model, are needed to confirm our current findings. Moreover, previous study showed that increased BDNF signaling within the NAc mediates susceptibility to CSDS-induced social avoidance (Krishnan et al., 2007, Cell). In our study, *Ghsr*^-/-^ mice displayed resistance to CSDS-induced anxiety and despair-like behavior, but susceptibility to CSDS-induced social avoidance, therefore examining BDNF levels in different brain regions may provide useful information regarding correlations between BDNF and mood disorders after CSDS.

BDNF/TrkB pathway is one of a few best-characterized signaling pathways underlying the pathophysiology of chronic stress and depression. The downstream signaling of BNDF/TrkB includes activation of Akt, GSK3β, ERK1/2, and PKC (Duman and Voleti, 2012). Interestingly, although GHS-R1a activation primarily engages excitatory Gq-dependent PLCγ/PKC/Ca^2+^ molecular cascades (Howard et al., 1996), it is also reported to link with other signal pathways including Raf/MEK/MAPK, PKA, PI3K/Akt/GSK3β, and etc. (Lodeiro et al., 2011; Ribeiro et al., 2014). GHS-R1a also exhibits an extremely high level of constitutive activity in the absence of bound ligand (Holst et al., 2003; Gagliardi et al., 2018). Since chronic stress elevated circulating ghrelin, and ghrelin/GHS-R1a signaling shares a majority of downstream molecular pathways involved in the pathophysiology of depression, we checked the activation of multiple signaling pathways in the hippocampus of GHS-R1a knock-out mice and their wild-type littermates exposure to CSDS, including activity of Akt, ERK1/2, JNK, CREB, and p38 MAPK. However, we did not found any difference between *Ghsr*^-/-^ mice and *Ghsr*^+/+^ control mice regarding signaling protein activities in the hippocampus, either at baseline state or after CSDS.

It is well proved that chronic or repeated exposure to social defeat stress results in abnormal activation of the immune system leading to a pro-inflammatory state (Takahashi et al., 2018). In particular, IL-6 has been linked to CSDS-related depression and anxiety. For example, susceptible C57BL/6 mice after CSDS, both male and female, exhibited higher IL-6 in serum compared to resilient mice, indicating that elevated IL-6 is a common mechanism mediating social stress susceptibility (Hodes et al., 2014; Takahashi et al., 2017). Increased circulating IL-6 was also observed in humans suffering from major depression (Hodes et al., 2014; Kiraly et al., 2017). Recent studies indicated that IL-6 is the most consistently elevated cytokine in the blood of MDD patients, therefore it may serve as a predictive biomarker and a potential target to treat depression in humans (Hodes et al., 2016). Consistently, we found that CSDS exposure increased IL-6 in serum of defeated *Ghsr*^+/+^ control mice, however, it did not affect the serum concentration of IL-6 in *Ghsr*^-/-^ mice undergoing same stress procedure (Figure 3). Therefore reduced IL-6 in serum of *Ghsr*^-/-^ mice may correlate to GHS-R1a deficiency-induced behavioral resistance to CSDS. It is worth mentioning that, in our study, the difference in BDNF expression between *Ghsr*^-/-^ mice and *Ghsr*^+/+^ control mice under CSDS was slight (although significantly different), while the difference in IL-6 expression between those two groups was rather dramatic. Therefore we predict that reduced IL-6 might have a bigger impact on the rescue effect of *Ghsr* mutation. How GHS-R1a deletion blocks elevation of IL-6 after CSDS exposure is uncertain yet. Previous studies have demonstrated that global GHS-R1a ablation protects against high fructose corn syrup- and aging-induced adipose inflammation (Ma et al., 2013; Lin et al., 2016). In particular, GHS-R1a ablation during aging was reported to shifts macrophages toward an anti-inflammatory state and reduces pro-inflammatory cytokine expression, including necrosis factor-α (TNF-α), IL-1β and IL-6, in adipose tissues. The same study also reported that GHS-R1a knockdown in macrophage RAW 264.7 cells directly decreases pro-inflammatory cytokines expression of TNF-α, IL-1β, and IL-6 (Lin et al., 2016). Moreover, our very recent study showed that myeloid-specific deletion of GHS-R suppresses high fat diet-induced neuro-inflammation in the hippocampus, likely due to decrease pro-inflammatory cytokines such as TNF-α, IL-1β and IL-6 (unpublished). All these findings suggested that GHS-R1a regulates both peripheral and central inflammatory response, and GHS-R1a deficiency is beneficial. Since current study was carried out in GHS-R1a global knockout mice, both peripheral and neuronal anti-inflammatory mechanisms may contribute to the observed effect of GHS-R1a deficiency on IL-6 expression.

Microglia pro-inflammatory activation is a landmark for stress exposure, especially chronic stress (Yirmiya et al., 2015; Wohleb, 2016). However, GHS-R1a was not detectable in microglia (Carniglia et al., 2017), therefore the effect of GHS-R1a ablation on IL-6 seems not directly associated with microglia activity. In addition, we did not find difference between *Ghsr^-^*^/^*^-^* and *Ghsr*^+/+^ mice regarding serum concentrations of leptin both at baseline state and after CSDS, suggesting that leptin signaling is not involved in GHS-R1a effect on inflammation and IL-6 expression. Mounting evidences also supports important roles for TREM (Triggering receptor expressed on myeloid cells) family, BDNF, astrocyte in the regulation of innate and adaptive immune responses (Huang and Reichardt, 2001; Sharif and Knapp, 2008; Norden et al., 2016). Unfortunately, current study did not start to explore those possibilities. Undoubtedly, further studies are required to elucidate the causal relationship or correlation between *Ghsr* expression and IL-6.

It is generally presumed that inflammation and BDNF interact negatively in the brain. Inflammation reduces neuroplasticity by down-regulation of BDNF, which may underlies pathophysiology of depression (Krishnan and Nestler, 2008; Schmidt et al., 2011). However, synergistic rather than antagonistic interactions between inflammation and BDNF were also reported in MDD patients. Specifically, serum BDNF was found to be positively associated with plasma IL-6 (but not TNF-α) in MDD patients, and IL-6 emerged as a robust positive predictor of BDNF only in MDD patients with melancholic features (Patas et al., 2014). Other study whereas reported weak correlation between levels of IL-6 and BDNF in depressed patients (Ninan et al., 2013). Interestingly, a recent study reported that elevated serum IL-6 was not only strongly associated with depression in cancer patients, but it was an independent, negative predictor of plasma BDNF level as well, while low BDNF in those patients was only associated with cognitive impairment but not depression (Jehn et al., 2015). Those findings seem not quite in accordance with the general hypothesis about inflammation and BDNF interaction in the pathophysiology of depression, nevertheless they all support the important role of elevated IL-6 in depression. The mechanisms by which IL-6 signaling may contribute to stress susceptibility and depression are unknown. Our study only began to explore the underlying mechanism for ghrelin/GHS-R1a-mediated pro-depression. More studies are needed to provide further evidence supporting the causal relationship or correlation between *Ghsr* expression, IL-6, BDNF and stress susceptibility.

## Conclusion

Our study demonstrated that GHS-R1a deficiency provides certain resistance to CSDS, the down-regulation of circulating IL-6 and the up-regulation of hippocampal BDNF may contribute to this process. Our findings thus support pro-anxiety and pro-depression effects of ghrelin/GHS-R1a signaling in response to chronic stress and mood disorders. Further studies are still needed to elucidate the underlying molecular and cellular mechanisms, such as how ghrelin/GHS-R1a system in chronic stress regulates expression of BDNF and IL-6.

## Ethics Statement

The animal protocols used here were approved by the Chancellor’s Animal Research Committee at Qingdao University (in accordance with National Institutes of Health guidelines).

## Author Contributions

LG and MN performed all experiments. JY, LL, and SL contributed to data analyses. YZ supervised the experiments. ZZ and YZ interpretated data and drafted the manuscript. YS and YZ revised the manuscript. All authors read and approved the final manuscript.

## Conflict of Interest Statement

The authors declare that the research was conducted in the absence of any commercial or financial relationships that could be construed as a potential conflict of interest.

## Supplementary Material

Figure S1 Chronic social defeat stress (CSDS) procedure and validation. **(A)** Illustration of the experiment procedure. Over 10 days, mice were introduced into the home cage of a different dominant CD1 mouse for 5 min daily. Behavioral tests began 1 day after defeat. Blood and brain tissues were collected immediately after behavioral tests. **(B)** Serum concentration of ACTH, *n* = 6 samples for each group. **(C)** Serum concentration of TNF-α, *n* = 7 for each group. **(D)** Serum concentration of IL-6, *n* = 7 for each group. **(E)** *Ghsr* expression in the hippocampus, *n* = 6 mice for *Ghsr*^+/+^ group and *n* = 7 for *Ghsr^-^*^/^*^-^* group. Unpaired *t*-test with Welch’s correction when necessary, ^∗^*P* < 0.05, *^∗∗^P* < 0.01 means significant difference. All data are shown as means ± SEM.

Figure S2 Comparing multiple peripheral and central biomarkers at baseline, non-stress state. **(A)** Serum levels and **(B)** hippocampal concentrations of multiple biomarkers. Serum samples, *n* = 4–7 for each group. Hippocampal samples, *n* = 7 for *Ghsr*^+/+^ group and *n* = 8 for *Ghsr*^-/-^ group. **(C)** Oxidative stress in the prefrontal cortex. *Ghsr*^+/+^, *n* = 5; *Ghsr*^-/-^, *n* = 6. Unpaired *t-*test with or without Welch’s correction. All data are shown as means ± SEM.
