# Supplementary figures and images for: GHS-R1a Deficiency Alleviates Depression-Related Behaviors After Chronic Social Defeat Stress
Source: Front Neurosci. 2019 Apr 17;13:364. doi: 10.3389/fnins.2019.00364 (PMC6478702; doi:10.3389/fnins.2019.00364)

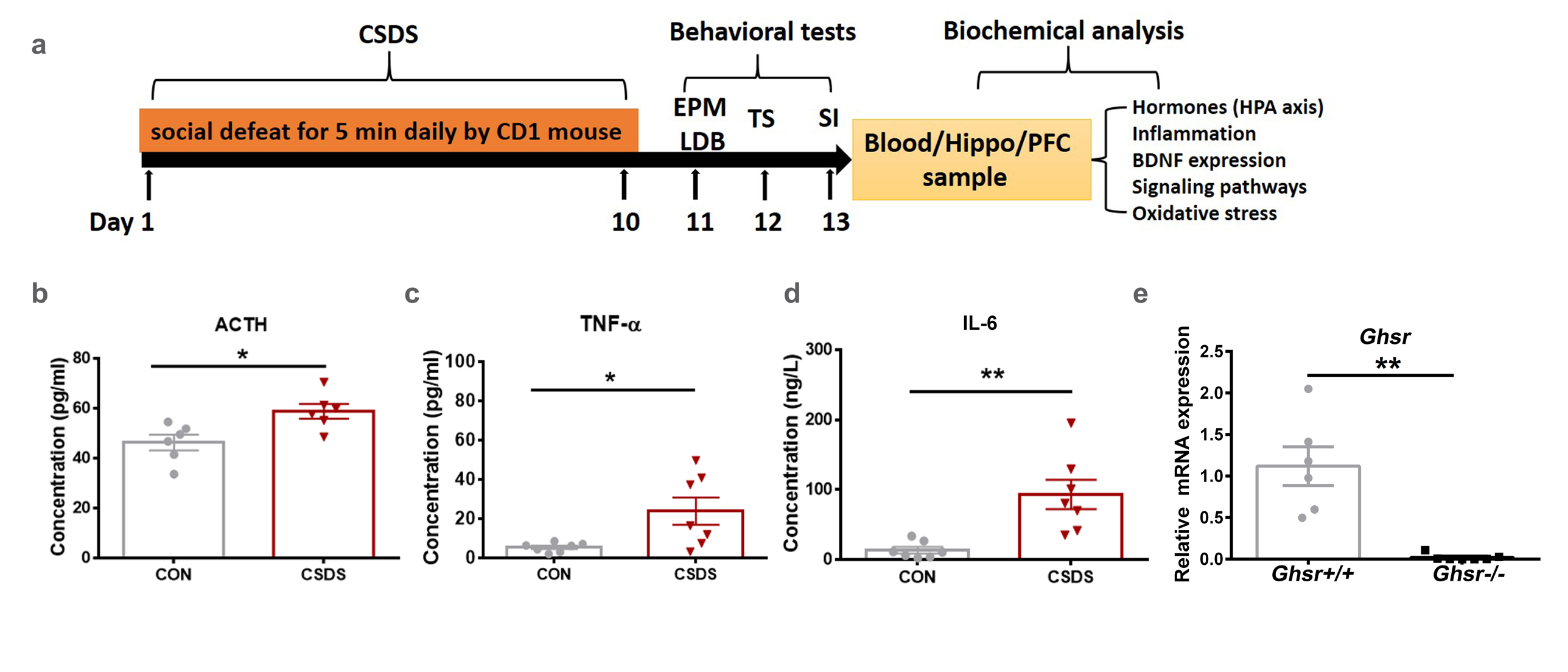

Supplement: Figure S1 — Chronic social defeat stress (CSDS) procedure and validation. (A) Illustration of the experiment procedure. Over 10 days, mice were introduced into the home cage of a different dominant CD1 mouse for 5 min daily. Behavioral tests began 1 day after defeat. Blood and brain tissues were collected immediately after behavioral tests. (B) Serum concentration of ACTH, n = 6 samples for each group. (C) Serum concentration of TNF-α, n = 7 for each group. (D) Serum concentration of IL-6, n = 7 for each group. (E) Ghsr expression in the hippocampus, n = 6 mice for Ghsr+/+ group and n = 7 for Ghsr-/- group. Unpaired t-test with Welch’s correction when necessary, ∗P < 0.05, ∗∗P < 0.01 means significant difference. All data are shown as means ± SEM. [file Image_1.JPEG]

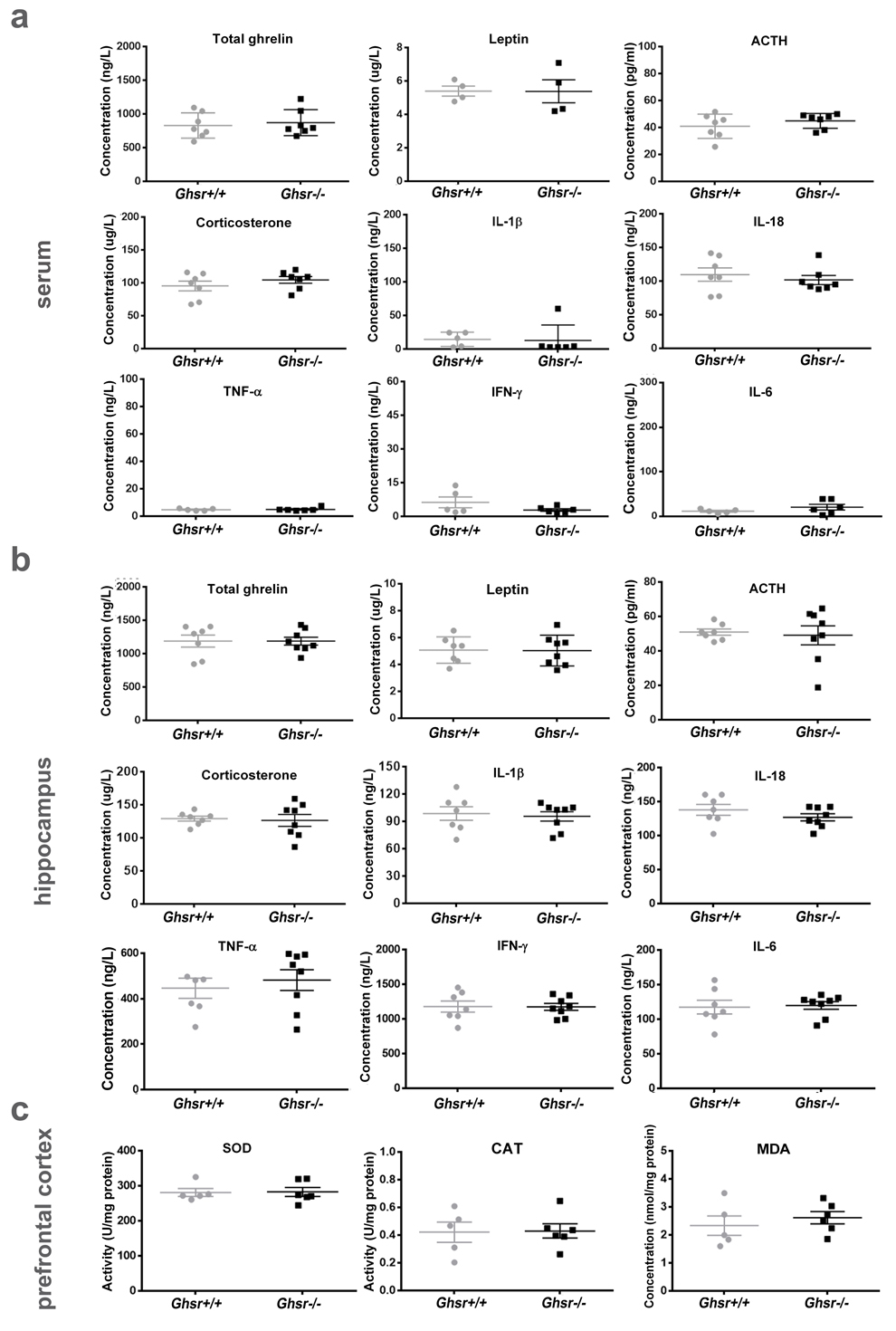

Supplement: Figure S2 — Comparing multiple peripheral and central biomarkers at baseline, non-stress state. (A) Serum levels and (B) hippocampal concentrations of multiple biomarkers. Serum samples, n = 4–7 for each group. Hippocampal samples, n = 7 for Ghsr+/+ group and n = 8 for Ghsr-/- group. (C) Oxidative stress in the prefrontal cortex. Ghsr+/+, n = 5; Ghsr-/-, n = 6. Unpaired t-test with or without Welch’s correction. All data are shown as means ± SEM. [file Image_2.JPEG]
